# Enucleation of Uterine Febroids

**Published:** 1873-06

**Authors:** 


### Enucleation of Uterine Fibroids.

Dr. T. Gaillard Thomas exhibited to the New York Obstetrical Society (Oct. 3d, 1872) a mass the size of a cocoanut, consisting of three fibroids which he had recently removed by enucleation. The woman, who was a patient of Dr. H. Moeller, had suffered for some months from excessive menorrhagia. When seen by Dr. Thomas, the uterus was found to be as large as at the fifth month of gestation; the cervix was found well dilated, and a large fibroid presenting, which, on examination, proved to be sessile, and attached posteriorly. The patient lying upon her back upon a table, Dr. Thomas proceeded to enuculate by cutting through the capsule of the tumour with scissors, and then insinuating a grooved steel sound under the capsule, which he separated as far as possible from its attachments. Traction was then resorted to, combined with powerful expression from above, which resulted in the extrusion of a large mass in about forty minutes; another mass was felt above the one extracted, which was in like manner removed; traction then being made upon the capsule, it came away, having attached to it a still smaller tumour. There was a very little hemorrhage. Opiates were given, and intra-uterine injections of carbolic acid were daily used. The patient did well for four weeks, though she now has a mild attack of phlegmasia dolens, from which she has suffered once before as a sequel to parturitition. This is the sixth case in which Dr. Thomas has resorted to enucleation, in all of which the recovery has been perfect. He has, however, lost two patients in the preparatory treatment by sponge-tents. Dr. Thomas considers the operation in its results more formidable than ovariotomy.—*Am. Jour. Obstetrics.*
—*Am. Jour. Med.*

